# Salinity controls rocky intertidal community structure via suppression of herbivory

**DOI:** 10.1002/ecy.70271

**Published:** 2025-12-08

**Authors:** Theraesa Coyle, Sandra Emry, Rebecca L. Kordas, Christopher D. G. Harley

**Affiliations:** ^1^ Department of Zoology University of British Columbia Vancouver British Columbia Canada; ^2^ Institute for the Oceans and Fisheries, University of British Columbia Vancouver British Columbia Canada; ^3^ Present address: Pacific Biological Station, Fisheries and Oceans Canada Nanaimo British Columbia Canada; ^4^ Present address: Hatfield Consultants North Vancouver British Columbia Canada

**Keywords:** environmental stress, herbivory, indirect effects, intertidal zone, salinity, Strait of Georgia, Salish Sea

## Abstract

Climate change impacts ecosystems directly through differences in species‐specific responses as well as indirectly through changes to the strength of species interactions. To predict how species will be impacted by ongoing environmental change, we need to better understand the relative roles of these direct and indirect effects. Salinity is a strong driver of ecological patterns and processes, and salinity regimes in coastal regions are expected to be altered by climate change through the intensification of the hydrological cycle and via climate‐driven shifts in the timing and strength of the spring freshet. We hypothesized that hyposalinity can indirectly affect the intertidal community by excluding a dominant guild of herbivores, limpets in the genus *Lottia*. To test this hypothesis, we (1) conducted intertidal diversity surveys in regions of high versus seasonally low salinity in the Strait of Georgia, British Columbia, (2) conducted laboratory salinity tolerance trials for two important grazers (*Lottia pelta* and *Lottia digitalis*) and one primary producer (*Ulva* sp.), and (3) experimentally manipulated the abundance of grazers in these two regions. We show that rocky intertidal shores from two regions of disparate salinity regimes are distinct in their intertidal communities: low salinity sites were composed primarily of *Mytilus trossulus*, *Fucus distichus*, and *Ulva* sp., whereas high salinity sites were dominated by *Chthamalus dalli*, *Lottia* spp., and *Mastocarpus* sp. Our laboratory trials confirmed that freshwater inputs experienced in the low salinity region resulted in hyposaline levels which exceeded the tolerance of *Lottia* spp., but not that of *Ulva* sp. Further, we show that by excluding grazers in high salinity sites, these communities more closely resemble those of the low salinity sites than those of other high salinity sites with grazers present. Together, these results demonstrate that the pattern of distinct estuarine intertidal communities in low versus high salinity regions in the Strait of Georgia may be largely driven by the indirect effects of freshwater inputs, mediated by salinity‐driven differences in herbivore population size and thus grazing pressure.

## INTRODUCTION

Understanding organisms' direct responses to abiotic stress is an important first step in explaining the distribution of species across environmental gradients in time and space. The tolerance of individual species to environmental stressors is frequently used to predict the likelihood that they will persist in the face of climate change (Louthan et al., 2021). However, predictions made solely on the basis of tolerance limits to stress and without reference to the influence of interacting species can lead to misleading results and fail to explain the observed distribution and abundance of species (Brooker et al., 2007; Davis et al., 1998; Hein et al., 2012; Van der Putten et al., 2010; Wallingford & Sorte, 2019; but see Thierry et al., 2021). A growing body of evidence suggests that environmental stressors impact community development and structure not only via direct effects on the physiology and survival of organisms, but also through indirect effects mediated by the interactions among species (Barton & Ives, 2014; Diamond et al., 2017; Kordas et al., 2011; Underwood, 1999). To better understand patterns of community composition and diversity and forecast how these patterns will shift due to ongoing environmental change, we need to integrate the direct effects of the environment on any given species with the indirect forcing that arises through environmentally driven shifts in trophic interactions, competition, and facilitation.

Environmental forcing is often mediated, or amplified, by changes in the strength of interspecific interactions (Kroeker & Sanford, 2022). Interactions, such as predation, herbivory, facilitation, and competition, are altered by both changes in the abundance of one or more interacting species, as well as changes in per capita interaction strength (Agüera et al., 2015; Kordas et al., 2011). Stressors that alter key ecological rates such as feeding through any of these pathways will therefore also have an indirect impact on prey species, which can have further cascading impacts on tertiary species (Paine, 1974). For example, tropical herbivorous fish have migrated into new habitats while tracking shifts in thermal isoclines, triggering a phase shift from a kelp‐dominated system to a rocky barren, with a coinciding loss of kelp‐associated diversity (Vergés et al., 2014). Further, environmental forcing can disproportionately affect some species over others; when “leverage species” (sensu Power et al., 1996) are particularly sensitive to changes in the environment, small alterations in the abiotic world can drive considerable change to community structure (Harley et al., 2006).

In addition to interspecific variation in environmental tolerance, differing abiotic conditions across a species range can result in divergent selection among populations, contributing to intraspecific variation in environmental tolerance (Sanford & Kelly, 2011). For example, despite gene flow among populations, an alpine grass species showed clear differentiation in fitness‐related traits along an elevation gradient (Gonzalo‐Turpin & Hazard, 2009). Likewise, juveniles of *Nucella lamellosa* originating from an area of high salinity experienced higher mortality under hyposaline stress, compared to juveniles originating from a region of low salinity (Covernton & Harley, 2020). Ultimately, the interplay between direct and indirect effects of abiotic factors at both a species and a population level will determine aggregate community‐level properties such as diversity and community structure.

We investigated the importance of direct and indirect environmental controls on estuarine rocky shores. Rocky intertidal shores are a unique system to study such ecological processes as they are highly dynamic in their physical environment, being at the interface of land and sea and exposed to the conditions of both with the rise and fall of the tides (Kunze et al., 2021). Fluctuations in abiotic conditions occur daily, seasonally, and in the long term in response to large‐scale geographical and climatological processes, further influencing the dynamic nature of intertidal communities (Hsieh et al., 2005; Stillman et al., 2025). Along with the natural variability in abiotic conditions, rocky shores are highly tractable, making these systems ideal for testing questions related to how species interactions mediate the impacts of abiotic stress on species composition.

In estuarine rocky intertidal ecosystems, salinity is one of the most important drivers of the performance of organisms at multiple scales of biological organization and thus has cascading impacts on population and community structure (Ritter et al., 2005). Exposure to fresh water can induce physiological stress responses in marine animals, including decreased heart rate, reduced hemolymph osmolality, and mortality (Chelazzi et al., 2001; Firth & Williams, 2009), as well as disrupt ecological processes such as feeding, activity, reproduction, and larval development rate (Cheung, 1997; Zimmerman & Pechenik, 1991). Similarly, decreased salinity levels have been found to reduce the survival, development, and settlement of marine larvae and subsequently influence adult distribution (Dineen & Hines, 1994; Starczak et al., 2011). Further, hyposaline conditions inhibit the growth and photosynthetic rate of many marine algal species (Connan & Stengel, 2011; Karsten, 2007), although several algal species have demonstrated a wide salinity tolerance range (Rath & Adhikary, 2005), as well as a capacity for local adaptation to low salinities (Nygård & Dring, 2008). As species and populations can have individualistic responses to salinity across a food web, salinity variation can result in changes in community structure and ecological processes that influence biodiversity (Hampel et al., 2009; Witman & Grange, 1998; Zacharias & Roff, 2001).

Here, we sought to understand the direct and indirect effects of spatial variation in salinity in the Strait of Georgia for intertidal algal‐herbivore interactions and the resulting community structure. We hypothesized that limpets (*Lottia* spp.) are disproportionately vulnerable to hyposalinity stress relative to other species, including resource species such as the green macroalga *Ulva* sp. We further hypothesized that differences in community structure between high and low salinity areas would be directly related to differences in limpet grazing pressure due to hyposalinity limits to limpet abundance and therefore only indirectly driven by salinity. To test these hypotheses, we combined laboratory salinity tolerance trials on limpets, specifically *Lottia pelta*, and the green macroalga *Ulva* sp., with observational surveys and a manipulative herbivore exclusion experiment at six sites, three each within an area of consistently high salinity, the Southern Gulf Islands, Canada, and in an area of seasonally low salinity, West Vancouver, Canada. First, we predicted a lower abundance of limpets in the periodically low salinity environments than in the consistently high salinity areas. Second, we predicted that any local adaptation to salinity in limpet populations, if present, would not be enough to overcome the minimum salinity level in West Vancouver to maintain a population level comparable to the rocky shores of the Southern Gulf Islands. Third, based on known effects of local grazers (Harley, 2006; Hesketh et al., 2021; Kordas et al., 2017) we predicted that a reduction in grazing pressure from limpets would result in an increase in algal cover and concomitant decrease in barnacle (*Chthamalus dalli*) cover in low salinity sites, or high salinity sites from which limpets had been experimentally excluded.

## MATERIALS AND METHODS

### Study system

**FIGURE 1:**
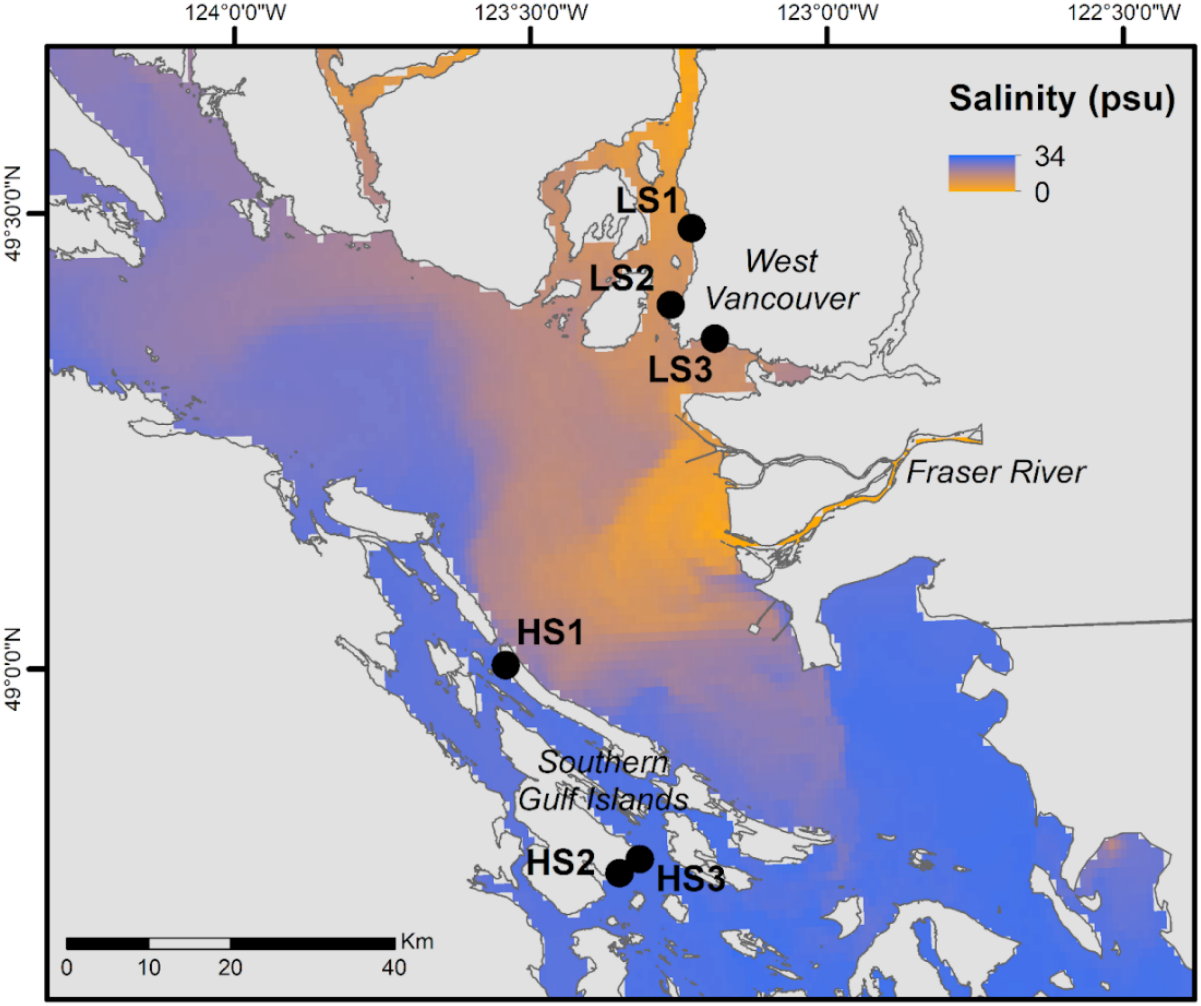
Map of the study region with LiveOcean modeled salinity for July 8, 2019 (MacCready et al., 2021). Low salinity sites are located in West Vancouver (LS1, LS2, and LS3). High salinity sites are located in the Southern Gulf Islands (HS1, HS2, and HS3).

This research took place in the Strait of Georgia, British Columbia. The 220‐km Strait is located between Vancouver Island and mainland British Columbia and is partially isolated from the Pacific Ocean by restricted flow through narrow channels around the northern and southern tips of the island. Freshwater inputs are dominated by the Fraser River, which regularly exceeds a mean outflow rate of more than 7000 m^3^/s in summer months during the freshet (ECCC 2012). The late spring peak in river discharge causes a corresponding reduction in sea surface salinity in the southern Strait of Georgia (Figure 1), with an annual drop from approximately 25 psu to less than 10 psu at coastal sites near the river mouth during peak discharge (Jarníková et al., 2022). This effect, however, declines with increasing distance from the Fraser River, with waters southwest of the Southern Gulf Islands maintaining salinities of 23 to 32 psu year‐round (MacCready et al., 2021). The contrasting salinity regimes on either side of the Strait make this system a unique and ideal environment for disentangling the direct and indirect effects of this environmental stressor on coastal marine communities.

**FIGURE 2:**
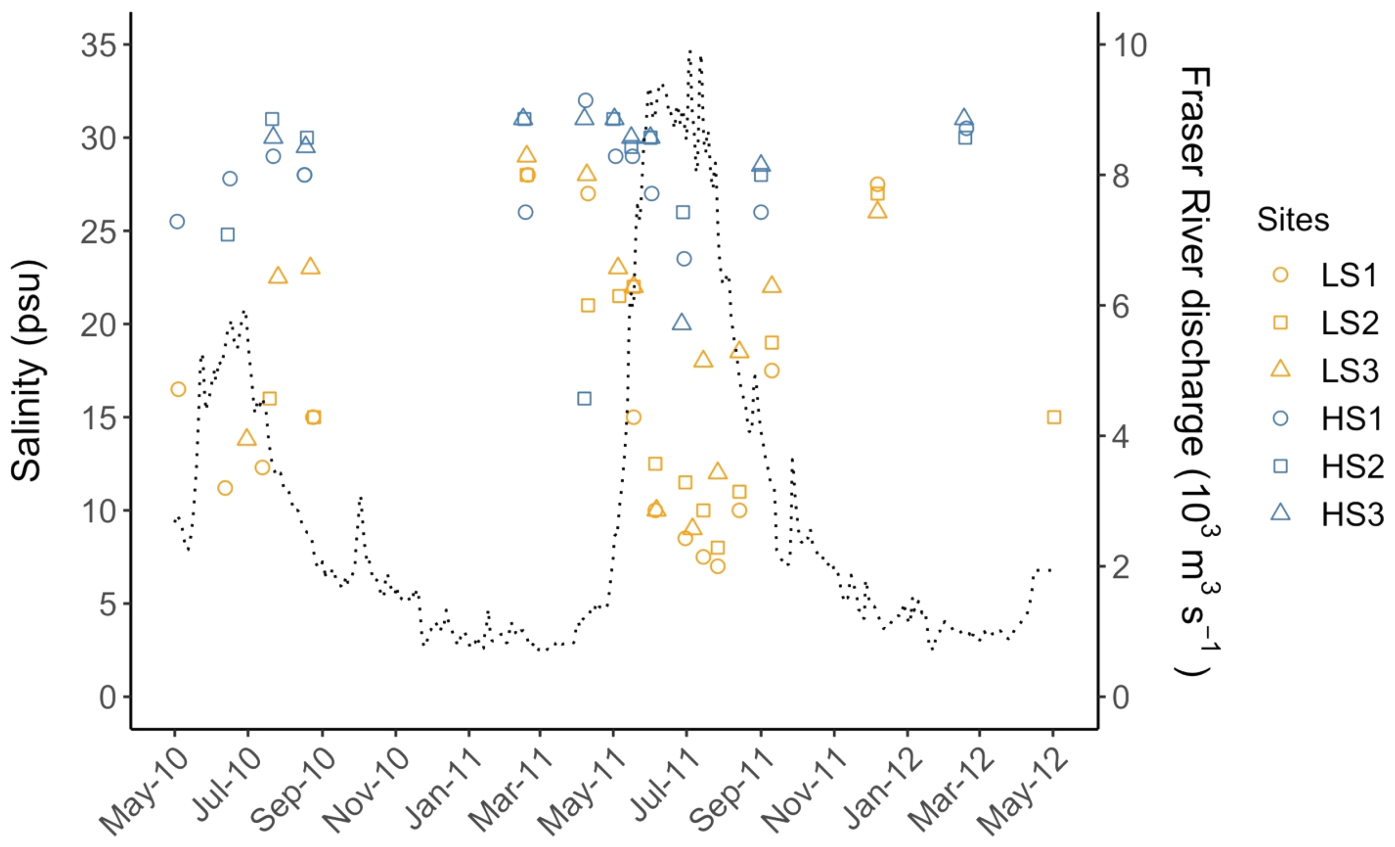
Measured surface salinity (psu) from sites in the Southern Gulf Islands and in West Vancouver, British Columbia. Dashed line indicates Fraser River discharge rate (10^3^ m^3^/s) measured at Hope, British Columbia (Environment Canada, 2012). Surface salinity for HS2, April 7, 2011, was influenced by heavy rainfall.

The field studies described here took place on the traditional, ancestral, and unceded territory of the Sḵwx̱wú7mesh (Squamish), xʷməθkʷəy̓əm (Musqueam), səlil̓ilw̓ətaʔɬ (Tsleil‐Waututh), Stz'uminus, Quw'utsun (Cowichan), Semiahmoo, scəw̓aθən məsteyəxʷ (Tsawwassen), SȾÁUTW̱ (Tsawout), Penelakut and Hwlitsum nations. We conducted field studies at three sites within each of two regions with contrasting salinity regimes: West Vancouver (low salinity sites LS1, LS2, and LS3) and the Southern Gulf Islands (high salinity sites HS1, HS2, and HS3; Figure 1). The Southern Gulf Island sites are located on the southwest side of the island chain and are not directly exposed to the Fraser River plume. Because of this, the HS sites remain at consistently high salinities year‐round, while the LS sites experience reduced salinities during summer (Figure 2). Water samples for salinity determination were collected immediately adjacent to the shore at a depth of ~15 cm and analyzed with a handheld refractometer. This was done during low tide visits approximately once a month throughout the summers of 2010 and 2011, as well as once for each site in the following winters. Sea surface temperatures in the two regions are comparable, ranging from 5.0 to 18.5°C in West Vancouver and 6.0 to 18.5°C in the Southern Gulf Islands (Fisheries and Oceans Canada, 2009). The tidal range is greater in West Vancouver, with extreme high tides reaching 4.7 m above Canadian chart datum (approximated as the lowest astronomical tide), compared to 3.4 m in the Southern Gulf Islands. All sites used in this study were composed of granitic rock except for HS1, which was sandstone. Areas selected for surveys and experiments were gently sloping (<40°) bedrock, with varying aspect (see Appendix S1: Table S1).

### Transect surveys

We conducted monthly surveys during low tide at each of the six study sites from May to August 2011. Because the tidal range differs between the two areas, we carried out surveys at the vertical height corresponding to approximately 30% immersion time. This occurs at 2.1 m in the Southern Gulf Islands and 3.0 m in West Vancouver. Ten meters of transect tape were laid across the rock face and eight randomly selected points were surveyed using a 25 × 25 cm quadrat. We counted all motile invertebrates and quantified sessile invertebrate and algal percent cover.

### Salinity tolerance experiments

#### Salinity tolerance and local adaptation of *Lottia pelta*

To determine the salinity tolerance of a common grazer, the limpet *L. pelta*, and whether the salinity tolerance was contingent on source population, we conducted an experiment with two populations: one from a high salinity site and one from a low salinity site. We collected *L. pelta*, 20 ± 5 mm in length, from HS1 (salinity of 27 psu) on June 2, 2011, and from LS3 (salinity of 10 psu) on June 6, 2011. While this offset in collection dates means that limpets from HS1 were acclimated to lab conditions for an additional 4 days, the duration of acclimation to salinity treatments was equivalent. Limpets from HS1 were randomly divided into eighteen 1 L Ziploc containers with mesh walls, for a total of six limpets in each. We placed each container inside of an aquarium containing seawater at 30 psu; the salinity of the water within these aquaria was lowered by 2.5 psu per day until a salinity of 20 psu was reached. Limpets were allowed to acclimate to this salinity for 10 days. We also randomly divided limpets from LS3 into an additional 18 containers (six limpets per container) and placed all containers into aquaria containing seawater at 10 psu, increasing the salinity at increments of 2.5 psu per day to 20 psu. These limpets were allowed to acclimate to the final salinity of 20 psu for 6 days. After the acclimation period was complete, we randomly arranged containers into 18 aquaria, all containing seawater at 20 psu, so that each aquarium contained one container of limpets from the high salinity site and one from the low salinity site. Aquaria were randomly assigned salinity treatments of 5, 8, 11, 14, 17, and 20 psu, with three aquaria for each salinity. Aquaria were covered, bubbled with compressed air, and placed in a recirculating sea water system to maintain a water temperature of 12°C. We lowered salinities at a rate of 3 psu every 30 min until the desired salinity was reached, and limpets remained submerged for 28 days. Each day, we examined limpets for signs of mortality, including tissue damage, discoloration, and rigidity; any dead limpets were removed. The experiment continued for 28 days, and limpets were not fed during this time.

#### Salinity tolerance of *Lottia* spp. with tidal emersion

To determine whether the salinity tolerance of limpets is influenced by the periodic emersion from hyposaline conditions experienced during low tides, we conducted a salinity tolerance experiment with *L. pelta* and *L. digitalis*, which incorporated a mimic of tidal exposure. See additional methods for this experiment in the supplementary material.

#### Salinity tolerance of *Ulva* sp.

We collected *Ulva* sp. from LS2 in West Vancouver, from a salinity of 28 psu on December 7, 2011. Approximately 5–6 g of blot‐dried *Ulva* sp. was placed into each of sixty‐five 1‐L plastic bottles. Each bottle was randomly assigned a salinity treatment between 0 and 30 psu at intervals of 2.5 psu and provided with compressed air. The 0 psu treatment contained only distilled water, while all other treatments contained combinations of filtered seawater at 31 psu and dechlorinated freshwater at 0 psu. Bottles were placed inside a flow‐through sea water system to maintain a water temperature of 12°C and provided 25 ± 5 μmol m^−2^ s^−1^ of continuous light. After 3 weeks, we blot‐dried and weighed all samples, and one sample from each treatment was randomly selected to be assessed for photosynthetic efficiency using a pulse amplitude modulation (PAM) fluorometer (Jr PAM, Heinz Walz GmbH). Light intensities were altered using a 240 W Fiber Optic Illuminator (6000‐1, Intralux) and screening filters. We dark‐acclimated samples for 1 h prior to measuring rapid light curves, fitted as:

$$\mathrm{ETR}={\mathrm{ETR}}_{\max}\times \tanh\left(\frac{\alpha \times \mathrm{PFD}}{{\mathrm{ETR}}_{\max}}\right)$$

where ETR_max_ is the maximum ETR, alpha is the apparent photosynthetic efficiency, and PFD is the photon flux density in μmol photons m^−2^ s^−1^ (Jassby & Platt, 1976).

### Herbivore exclusion experiment

We manually cleared all organisms at seven subsites within each of the six study sites. Similar to the transect surveys, these plots were placed at a tide height corresponding to approximately 30% immersion time. Each subsite included a limpet inclusion, exclusion, and control plot. Inclusions and exclusions were formed by securing two copper fences, 2.5 cm high and 28.5 cm in diameter, to the rock face using Quickcrete quick drying cement. Copper enclosures/exclosures of this type are effective barriers to limpets (Harley, 2002) and partial barriers to periwinkles (Harley, 2006). We marked one circular plot within each area, also 28.5 cm in diameter, with stainless steel bolts to serve as a control. The positions of control and treatments were randomized within each subsite. We did not include copper controls in this study, as previous work has shown that partial copper treatments lead to partial effects which are difficult to interpret (Johnson, 1992). Ultimately, local salinity levels exceeded the lower tolerance limit of limpets in the low salinity sites, and the inclusion plots were not effective at retaining *L. pelta*. Because of this, we only analyzed exclusion and control plots at each subsite. We maintained the exclusion treatments by removing limpets, along with any other grazers found inside the rings, every 2 weeks. We censused plots once per month during low tide, from May to August. We used a 10 ✕ 10 cm quadrat to count motile invertebrates and barnacles and estimate percent cover of algae and mussels within each treatment. Salinity samples were taken at each sampling event and measured using a refractometer (S/Mill‐E, Atago Inc.).

### Statistical analyses

All analyses were completed using R version 4.1.2 (R Core Team, 2020). To test whether salinity regimes between our study areas were different, we performed a student's two‐sample *t*‐test on the 10th percentile of salinities measured across 2 years at each of the six sites.

To confirm our prediction that limpets are more prevalent in the high salinity sites, we modeled the total abundance of all limpet species identified in the transects with a negative binomial distribution using the MASS package (v 7.3‐64). We first attempted to include site as a random effect nested within the salinity region, but this model failed to converge. Instead, we fit site and month, as well as the interaction between the two, as fixed effects. In addition, to confirm our prediction that *Ulva* sp. is more common in the low salinity sites, we modeled the percent cover of *Ulva* sp. with a tweedie distribution using the glmmTMB package (v. 1.1.11). This model included month and salinity region as fixed effects, with site nested within region as a random effect. We tested the fit of these models with the car package (v 3.1‐3), using a type III sums of squares Wald Chi‐squared test.

We analyzed community data from the transect surveys using the *vegan* package (v2.5‐7; Oksanen et al., 2017). Species abundances were fourth root transformed to reduce the influence of highly abundant species and then standardized by the range of species abundance to account for the presence of both percent cover and raw abundances measured in a single site. We conducted a PERMIDISP test with 999 permutations, to ensure that the levels of dispersion among the groups are equivalent with the data transformation utilized. To test the hypothesis that species composition differs between salinity regions, we performed a permutational multivariate analysis of variance (PERMANOVA) on the transformed data. We also included a main effect of month (May, June, July, August) and an interaction term of salinity region crossed with month to determine whether species composition varied similarly across all summer months. To respect the dependence of sampling blocks within a site, we restricted the permutation scheme such that all quadrats along a transect were always permuted together. In doing so, 999 permutations were run on a matrix of Bray–Curtis dissimilarities. We then visualized the relationship between species composition and the explanatory variables, salinity region and month, with Canonical Analysis of Principal coordinates (CAP; Anderson & Willis, 2003). Finally, we conducted a Similarity of Percentages (SIMPER) analysis to investigate which species contributed most to the observed differences in salinity regions and/or months.

To test whether survival of *L. pelta* populations originating from a low and high salinity region differs along a gradient of salinity levels, we conducted a survival analysis with the *survival* package (v3.3‐1; Therneau, 2023). We modeled the probability of survival with the Kaplan–Meier method, which is a nonparametric method to estimate survival probability from observed survival events. We then used these model fits to calculate the restricted mean survival time (RMST), for each site by population combination. Using the RMST is an alternative to proportional hazards modeling, which requires a constant relative hazard over time that is not met with our data. To determine differences in net productivity of *Ulva* sp., we used a least squares regression to analyze the change in biomass before and after the treatments, as well as ETR_max_ at the end of the experiment.

Due to a lack of recruitment in spring months and a late summer heat wave in August 2011 that resulted in the die‐off of many species during our herbivore exclusion experiment, we analyzed community data in the penultimate sampling point of July only. We used the same methods as described above in the transect surveys subsection. We restricted permutations similarly, keeping each paired control and exclusion plot within a subsite together. We then conducted a SIMPER analysis to investigate which species contribute the most to observed differences among salinity regions, grazer treatments and the interaction between the two. Prior to running the SIMPER analysis, we removed grazers from the site‐species matrix, to ensure that our analysis does not identify a species that differed among treatments due to experimental manipulation. Finally, we fit generalized linear models, using the *glmmTMB* package (v1.1.2.3: Brooks et al., 2017), to the abundance of two species which were consistently identified as the most influential taxa in the SIMPER analysis, and had abundance patterns that were markedly altered by our experimental design: *Ulva* sp., and the barnacle *Chthamalus dalli*. Both models included an interaction between the two fixed effects, region and treatment, and site as a random effect nested within salinity region. To model percent cover of *Ulva* sp., we first attempted to fit a model with a beta error distribution, but this model failed to converge; therefore, we used a tweedie error distribution with variance among regions modeled independently. We modeled abundance of *C. dalli* with a negative binomial error distribution, with a zero‐inflation parameter. Both models were fitted with a type III sums of squares Wald Chi‐squared test. We checked model diagnostics with the *DHARMa* package (v 0.4.5; Hartig, 2022), by running K‐S tests, Levene's test and plotting scaled residuals against each predictor variable.

## RESULTS

### Abiotic conditions

Salinity varied in the Strait of Georgia over both space and time. Salinity decreased as a result of increasing Fraser River discharge in spring and summer in both regions but these seasonal differences were much more pronounced in the low salinity region compared to the high salinity region (Figure 2). The tenth percentile of salinity measured from 2010 to 2012 in the LS sites was 9.6 psu (± 0.9 SE), over 15 psu lower than the tenth percentile of 26 psu (± 0.9 SE) measured in the HS sites (*t*_4_ = −13.35, *p* < 0.001).

### Transect surveys

**FIGURE 3:**
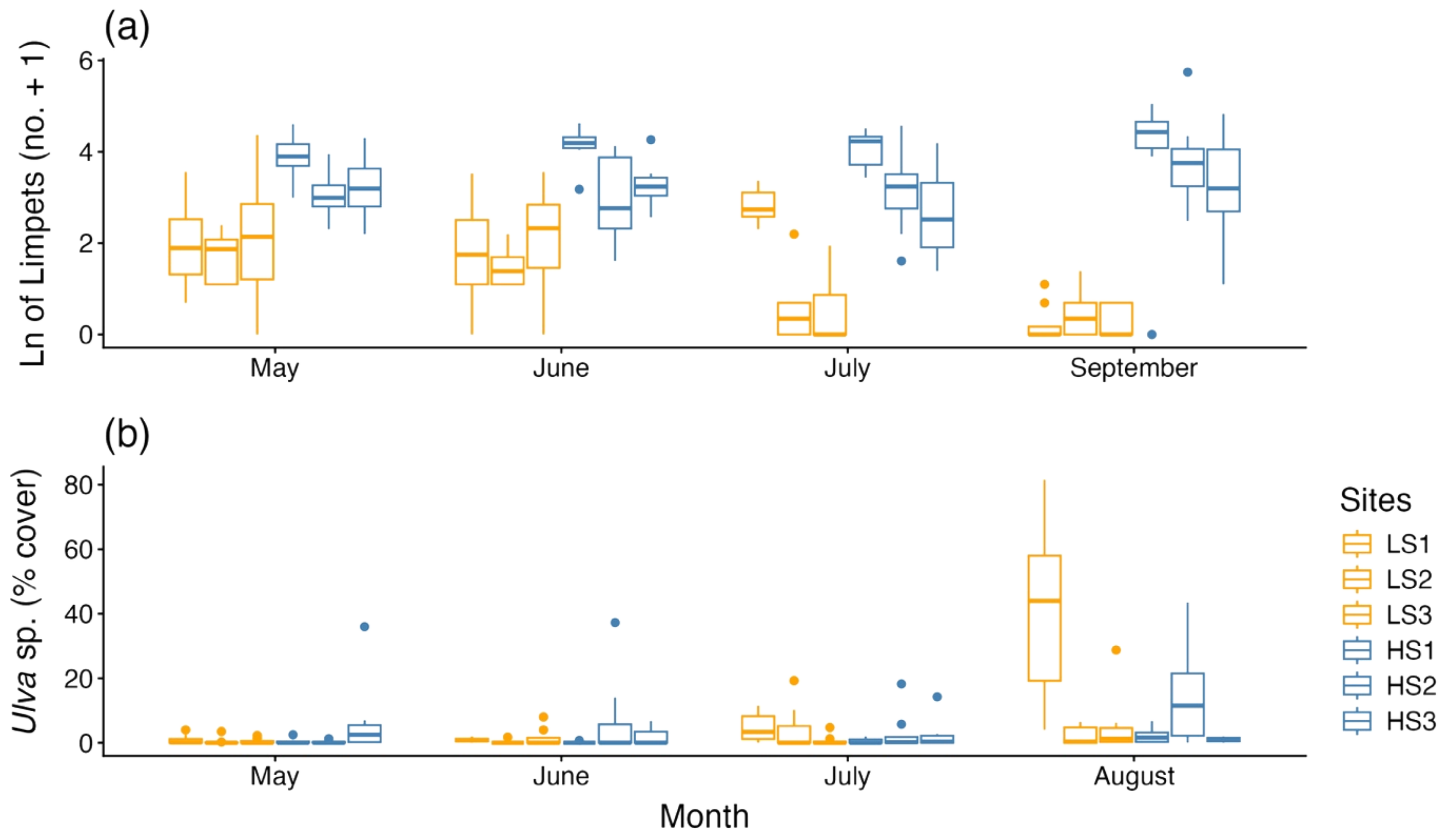
Boxplots of (a) total number of all limpet species and (b) *Ulva* sp. percent cover quantified from transects conducted in the intertidal zone of a low salinity region, West Vancouver (LS1, LS2, LS3), and a high salinity region, the Southern Gulf Islands (HS1, HS2, HS3) during the summer of 2011.

Limpet densities were consistently higher in the high salinity region (salinity region, χ^2^ = 6.51, df = 1, *p =* 0.01). Across all months, the high salinity region had an average of 44.5 limpets (±1.3 SE) per transect, whereas the low salinity region only had 6.9 limpets (±4.3 SE). As summer progressed, limpets became less prevalent in the low salinity region, increasing the difference in limpet densities between the two regions (month × salinity region, χ^2^ = 65.54, df = 3, *p <* 0.001; Figure 3a). *Ulva* sp. coverage varied month to month (month, χ^2^ = 41.76, df = 3, *p <* 0.001; Figure 3) but was only higher in the low salinity region in certain months (month × salinity region, χ^2^ = 11.9, df = 3, *p =* 0.007; Figure 3b). Photographs from these two regions, taken in the years before and after 2011 (when our transects were conducted), suggest that we may have surveyed during a year when Ulva abundance was lower than usual (Appendix S1: Figure S1).

**FIGURE 4:**
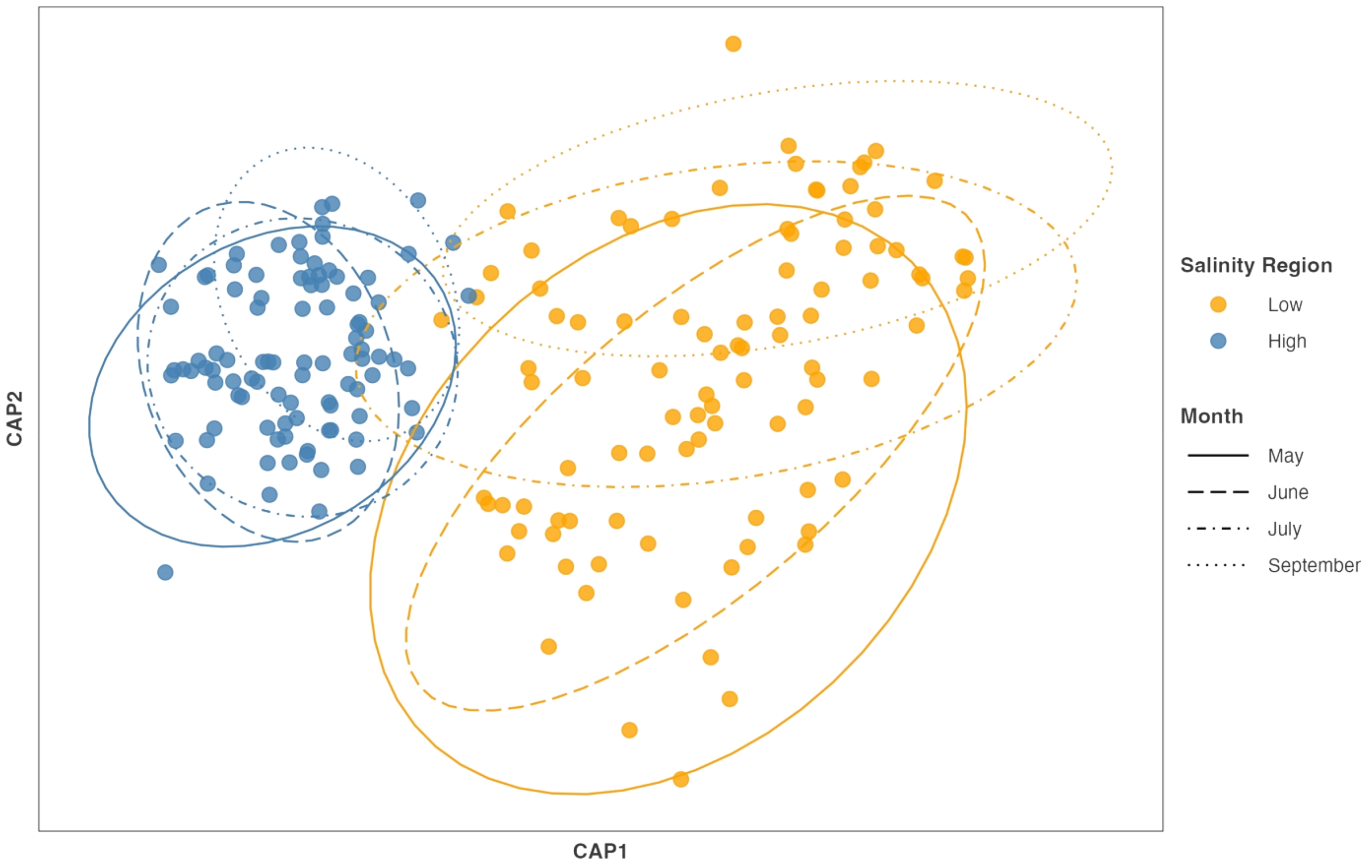
Canonical Analysis of Principal coordinates (CAP) of a Bray Curtis dissimilarity matrix based on species abundance data collected in May to August 2011, from transect surveys in regions of low and high salinity: West Vancouver (LS1, LS2, and LS3) and Southern Gulf Islands (HS1, HS2, and HS3). We analyzed the data with a PERMANOVA and show that salinity region (*F* = 99.75, *p* < 0.001), month (*F* = 5.57, *p* < 0.001), and the interaction between the two (*F* = 2.625, *p* = 0.001) all had a significant impact on community composition.

Communities belonging to low or high salinity regions had substantial differences in community composition from each other (PERMANOVA, *F*_1,184_ = 99.75, *p* = 0.001; Figure 4). Additionally, low salinity sites changed through time whereas the high salinity sites remained similar (region × month, PERMANOVA, *F*_3,184_ = 2.25, *p* = 0.001; Figure 4). Different months of summer also had significantly different communities across salinity regions (PERMANOVA, *F*_3,184_ = 5.57, *p* = 0.001; Figure 4). Dispersion did not differ among salinity groups or months (PERMDISP, *F*_7,184_ = 3.29, *p* > 0.05; Appendix S1: Table S2). The following species contributed the most to differences between salinity groups: the bay mussel *Mytilus trossulus*, the acorn barnacles *Balanus glandula* and *Chthamalus dalli*, the brown alga *Fucus distichus*, and the *Petrocelis* phase of the red alga *Mastocarpus* sp. LS sites were composed of more *M. trossulus* and *F. distichus*, while HS sites were composed of more *B. glandula*, *C. dalli*, *Mastocarpus* sp., and *Lottia paradigitalis* (SIMPER; Appendix S1: Table S3). Species‐specific responses are shown graphically in Appendix S1: Figure S2a–h.

### Salinity tolerance experiments

#### Salinity tolerance and local adaptation of *Lottia pelta*

**FIGURE 5:**
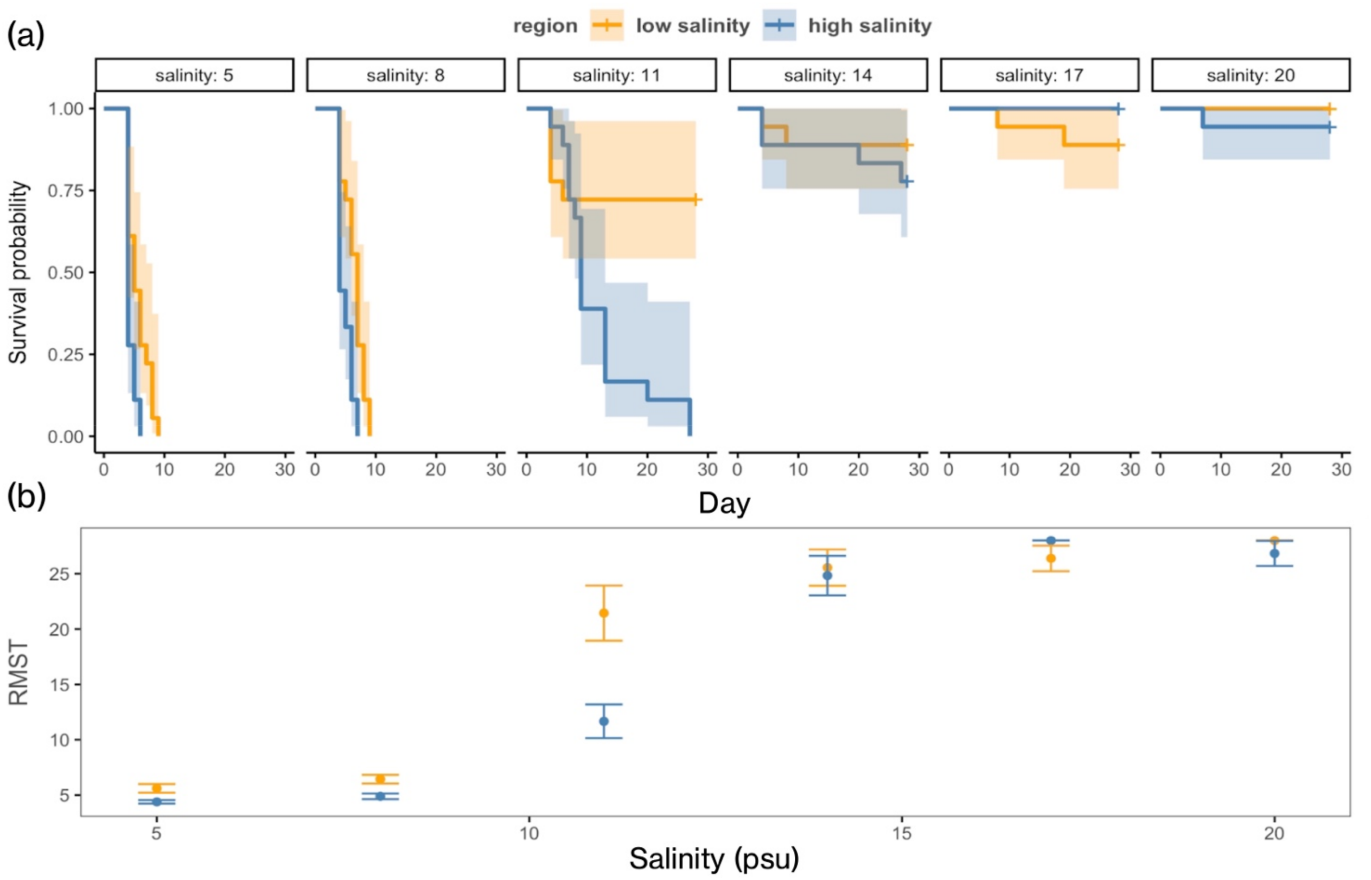
*Lottia pelta* survival from HS1 and LS3 at a range of salinity levels. (a) Kaplan–Meier survival curves for each of the population and salinity treatments over the 28‐day experiment. Survival probability is indicated on the *y*‐axis, where 1.0 is equivalent to 100% survival; the shaded area indicates 95% confidence intervals. (b) Restricted Mean Survival Time (RMST) calculated for the duration of the 28‐day experiment. Error bars represent standard error.

Survival of *L. pelta* was strongly reduced by low salinity, although the impacts of salinity differed between high and low salinity populations (Figure 5). Limpets could only survive for a few days at the two lowest salinities tested (5 and 8 psu), but the low salinity population had a higher RMST (5.6 ± 0.4 days at 5 psu and 6.4 ± 0.4 days at 8 psu) than the high salinity population (4.4 ± 0.2 days at 5 psu and 4.9 ± 0.3 days at 8 psu). The difference in survival among populations was amplified at 11 psu, where most low salinity limpets survived for the duration of the experiment with survival time estimated as 21.4 ± 2.5 days, as compared to 11.7 ± 1.5 days for the high salinity population. Limpet survival was uniformly high at salinities of 14, 17, and 20 psu, and populations did not differ in their RMST at these salinities.

#### Salinity tolerance of Lottia spp. with tidal emersion

Neither the survival of *L. pelta* nor *L. digitalis* was affected by daily tidal emersion (Appendix S1: Figure S3). For example, the mean survival time for *L. digitalis* in 10 psu seawater was 22.4 days (±1.3 days) when emersed for 8 h each day, compared to 22.8 days (±1.10 days) when immersed for the duration of the experiment. Similarly, the mean survival time for *L. pelta* in the 10 psu seawater, daily emersion treatment was 17.6 days (±2.5 days) compared to 21.2 days (±1.5 days) when fully immersed (see Supplementary material for detailed results).

#### Salinity tolerance of *Ulva* sp.

**FIGURE 6:**
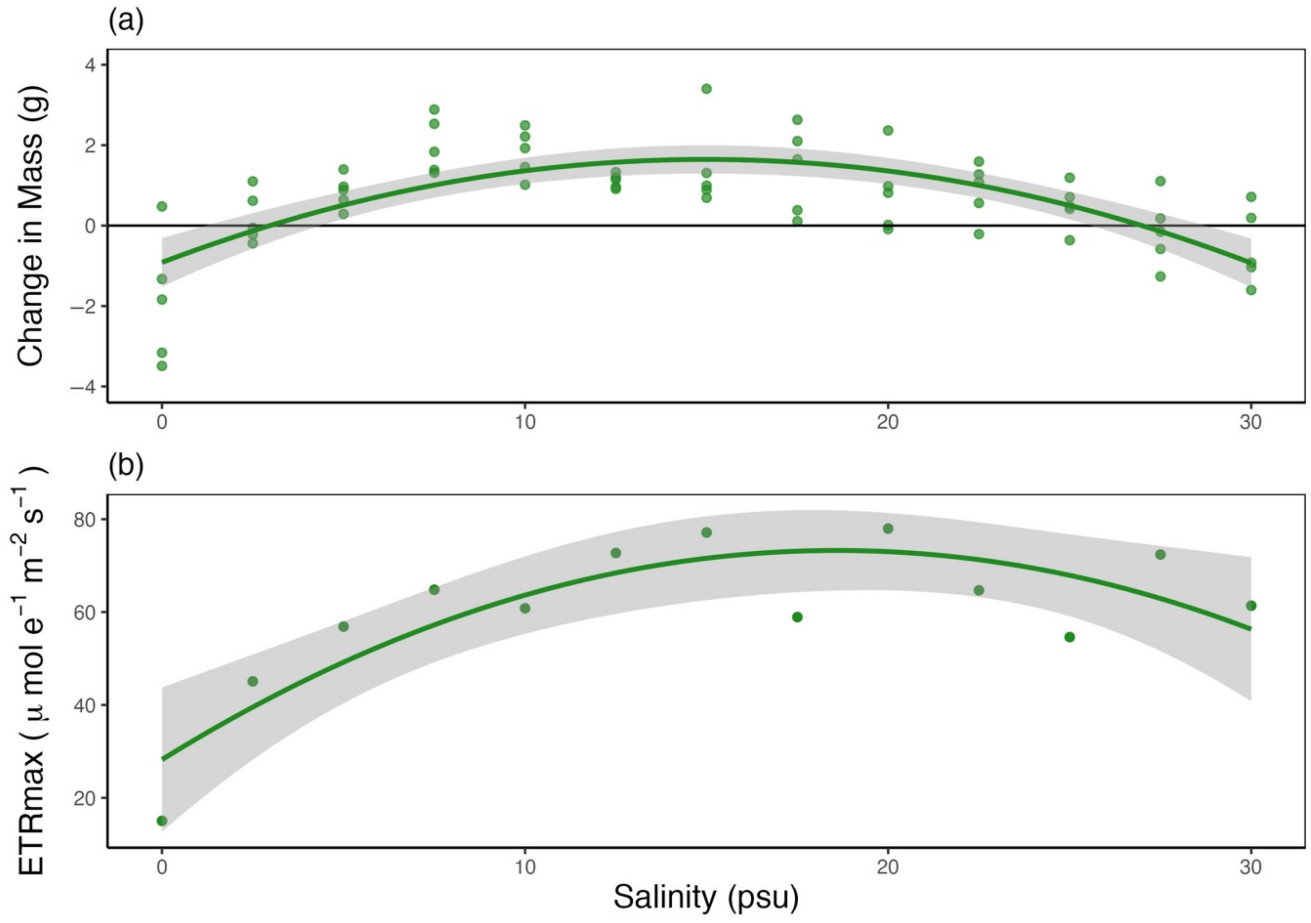
*Ulva* sp. salinity tolerance (a) Change in mass (g), (least squares regression, *y* = −0.915 + 0.342*x* − 0.0114*x* ^2^, *R* ^2^ = 0.48, *p* < 0.001) and (b) maximum electron transport rate (ETR_max_), (least squares regression, *y* = 28.2 + 4.84*x* − 0.13*x* ^2^, *R* ^2^ = 0.71, *p* = 0.002). The data were fitted to a second order polynomial, and the shaded area depicts 95% confidence intervals.

The net productivity of *Ulva* sp. was significantly and unimodally related to salinity (Figure 6a; *R* ^2^ = 0.48, *p* < 0.001), with the greatest gain in mass at 15 psu and net losses at both 0 and 30 psu. ETR_max_, a proxy for photosynthetic capacity, showed a similarly significant unimodal relationship (Figure 6b; *R* ^2^ = 0.71, *p* = 0.002), with a maximum value at 20 psu and minimum at 0 psu.

### Herbivore exclusion experiment

**FIGURE 7:**
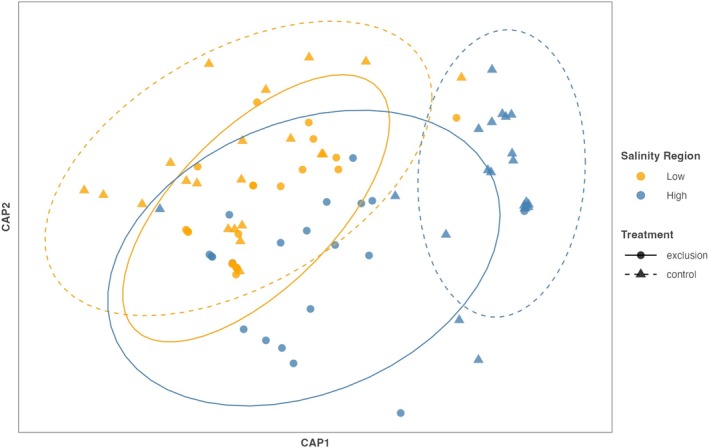
Canonical Analysis of Principal coordinates (CAP) based on a Bray–Curtis dissimilarity matrix of species abundance data collected in July 2011, from herbivore experimental plots (herbivore exclusions and control plots) at low (West Vancouver) and high salinity sites (Southern Gulf Islands). Both the grazer manipulation treatment (PERMANOVA, *F* = 3.41, *p* = 0.002), and the interaction between grazers and salinity region had a significant impact on community structure (PERMANOVA, *F* = 1.52, *p* = 0.044).

Herbivore exclusion had little influence on community structure in the low salinity region but had a large effect in the high salinity region (Figure 7). Notably, the communities in the high salinity herbivore exclusion plots were more similar to low salinity communities (with or without herbivore exclusion fences) than to high salinity plots with herbivores; salinity region had a significant effect on community structure (PERMANOVA, *F*_1,80_ = 23.68, *p* = 0.001; Figure 7), as did treatment (PERMANOVA, *F*_1,80_ = 8.23, *p* = 0.001) and the interaction between salinity region and treatment (PERMANOVA, *F*_1,80_ = 9.18, *p* = 0.001). Dispersion was unequal among salinity groups (PERMDISP, *F*_1,82_ = 17.3, *p* = 0.008; Appendix S1: Table S4), as well as among treatment groups (PERMDISP, *F*_1,82_ = 7.5, *p* = 0.001; Appendix S1: Table S4).

**FIGURE 8:**
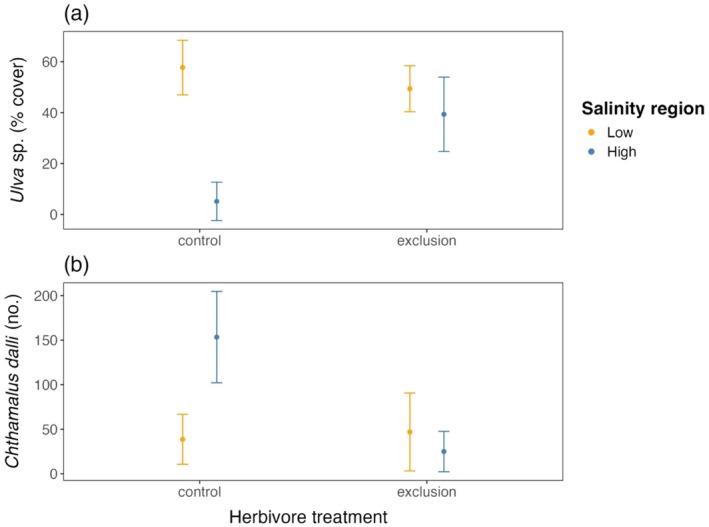
Mean abundance of (A) *Ulva* sp. and (B) *Chthamalus dalli* in the herbivore exclusion experiment in three low salinity (Southern Gulf Islands) and three high salinity sites (West Vancouver), censused in July 2011, 2 months after treatment establishment. Error bars represent standard error.

Among the high salinity sites, the effect of grazing on composition was primarily due to differences in *Ulva* sp. and the barnacles *B. glandula* and *C. dalli* (SIMPER, Appendix S1: Table S5). The same three species also had the highest contribution to between group differences in the high salinity and low salinity plots with grazers. Excluding grazers differentially affected the abundance of *C. dalli* and *Ulva* sp. across salinity regions. There was a consistent pattern of more *Ulva* sp. in the low salinity sites than in the high salinity sites, regardless of grazer treatment (χ^2^ = 20.06, df = 1, *p* < 0.001). However, excluding grazers had a strong impact on *Ulva* sp. cover in the high salinity sites, such that the grazer exclusion plots in the HS sites had similar *Ulva* sp. cover to all the plots in the LS sites (grazer × salinity, χ^2^ = 14.99, df = 1, *p* < 0.001; Figure 8a). Conversely, excluding grazers had the opposite effect on *C. dalli* barnacle abundance. Grazer‐excluded plots in the HS sites had an order of magnitude fewer *C. dalli* barnacles than the control plots in the same region (grazer × salinity, χ^2^ = 17.1, df = 1, *p* < 0.001; Figure 8b), resulting in *C. dalli* densities in high salinity grazer exclusion plots being similar to those in both grazer treatments in the low salinity region. Results for the other species are shown graphically in Appendix S1: Figure S4a–f.

## DISCUSSION

As climate change continues to alter the biophysical world and places increasing pressure on ecosystems, understanding the mechanistic link between the abiotic environment and the resultant patterns of community composition is imperative. A multitude of environmental drivers, which include salinity among others, are shifting in space and time due to anthropogenic effects (IPCC, 2023). The combination of gradients in abiotic drivers and resultant shifts in community interaction webs can create complex changes in community structure and ecosystem function (drought: Amundrud & Srivastava, 2016; Chase & Knight, 2003; precipitation: Barton & Ives, 2014; warming: Robinson et al., 2017; snowpack: Brodie et al., 2012). Although the correlation between the abiotic environment and species abundance and distribution is well studied (Walther et al., 2002), the extent to which this pattern is driven directly by changes to performance and survival or indirectly by changes to species interactions is not fully known (Bertness et al., 1999; Blois et al., 2013; Brown et al., 2001). Empirical studies that disentangle the impacts of direct environmental stress and the indirect effects of modified species interactions in natural communities are rare.

There were distinct differences in both salinity regime and intertidal community structure in our two study regions. As freshwater discharge from the Fraser River increased in the spring, salinity decreased in both LS and HS sites, but this reduction was much more pronounced in the LS sites, as these sites were in closer proximity to the river mouth. In the LS region, the average tenth percentile of salinity was 9.5 psu, which is in stark contrast to the 10th percentile of 26 psu experienced in the HS sites. Species driving the differences in the community composition among salinity regions consisted of a greater abundance of *M. trossulus* and *F. distichus*, and in some places *Ulva* sp. (e.g., late summer in the transects; the control plots in our experimental manipulations) in the LS sites, and barnacles, red algae, and grazers like the limpet species *L. paradigitalis* and *L. pelta* in the HS sites. Interestingly, community composition changed as the summer progressed in the low salinity region but not in the high salinity region. This context‐dependent shift in community structure may correspond with differences in the seasonal variation in salinity in the two regions (i.e., strong seasonal swings in salinity in the LS sites and only weak seasonal changes in the salinity of HS sites), which are driving shifts in species presence or abundance.

Our results align with other studies that found similar patterns of distinct communities associated with distance from freshwater sources such as riverine input or glacial melt (Giménez et al., 2010; Hossain et al., 2019; McCabe & Konar, 2021). This pattern of disparate communities among habitats that lie along a gradient of abiotic stress is not unique to salinity alone; documented differences in species composition have also been shown for freshwater phytoplankton communities along a thermal gradient, plant communities along an elevation gradient, and butterfly species in areas of low and high rainfall, to name a few (Beirão et al., 2017; Hailemariam & Temam, 2020; Ikram et al., 2022). Despite the importance of identifying differences in species composition among regions of varying environmental conditions, teasing apart the relative importance of whether indirect or direct impacts of abiotic stress in driving these patterns is challenging for purely observational datasets.

The divergent pattern in limpet abundance between regions over summer likely reflects the influence of the low salinity riverine output. Limpets are osmoconformers and thus are unable to regulate their extracellular osmolality in response to changes in their environment, leading to deleterious effects on both physiological and behavioral responses, and ultimately survival (Chaparro et al., 2008; Firth & Williams, 2009; Morritt et al., 2007). In the HS sites, limpets were able to survive a small seasonal decrease in salinity and maintain a constant abundance throughout summer. In contrast, limpets in the LS sites nearly disappeared as surface salinity approached freshwater levels, likely as a result of mortality related to osmotic stress.

Additional support for our hypothesis that regional differences in the abundance of limpets are driven by salinity tolerance comes from our lab assays of hyposalinity tolerance limits. Here, we show that limpet survival is strongly compromised below 11 psu in populations originating from both regions, but to a greater degree for limpets from the HS site. While limpets are broadcast spawners with moderate dispersal ability and are thus likely to possess a certain degree of population connectivity, even species with dispersive planktonic larval stages can show signatures of local adaptation when faced with strong enough selective pressures (Sanford & Kelly, 2011). Despite a propensity for local adaptation or acclimatization, limpets—regardless of population of origin—were not able to survive exposure to the full range of salinities potentially encountered in the LS sites. In general, this intolerance to low salinity is in accordance with previous research on intertidal gastropods (e.g., Covernton & Harley, 2020; Wilson et al., 2009).

Based on our results, limpet populations are expected to experience substantial mortality after less than a week of exposure to salinities less than 11 psu. Interestingly, while limpet abundance was exceedingly low in the LS sites over the summer, some individuals were able to persist in this hyposaline environment, at levels as low as 7 psu. Our lab experiment shows that this likely is not a result of daily aerial emersion during low tides, but there are other potential, non‐mutually exclusive explanations as to why some limpets could survive in the LS sites. For example, there are likely periods of elevated salinity due to tidal dynamics or wind‐driven mixing that were not captured by our salinity sampling. Alternatively, source‐sink dynamics may be at play, where populations are being replaced annually through recruitment from high salinity source populations.

Salinity‐driven differences in limpet abundance had cascading consequences for community structure across the salinity gradient. When grazers had been excluded from plots in the HS sites, communities resembled the LS sites to a remarkable degree, with more *Ulva* sp. and fewer barnacles, particularly *C. dalli*. Although the role of herbivory in structuring communities has been documented in several other systems besides rocky shores, such as the role of Canada geese in wetlands or Rocky Mountain elk in high‐elevation riparian areas (Jobe et al., 2022; Parsons et al., 2012), it seems to be especially strong in marine systems where herbivore gain or loss can result in striking community shifts (Bellwood et al., 2004; Hughes, 1994; Ledlie et al., 2007; Poore et al., 2012; Shurin et al., 2006). Herbivores can thus impact successional trajectories and habitat complexity and alter the strength of competition in communities. Unfortunately, we were unable to determine the effects of grazing in the LS sites, as the poor salinity tolerance of limpets meant that they were unable to survive in the inclusion plots. Had limpets been able to survive and feed in these inclusion plots, we would predict a decrease in *Ulva* sp. cover, which would likely facilitate an increase in *C. dalli* and red algae in the LS sites, as these species were already present and therefore have a salinity tolerance that allows at least some population persistence here. Ultimately, we need a better understanding of the salinity tolerance of these other species to understand the full effects of limpet grazing in this system.

*Ulva* sp. often has a strong role in the successional trajectories of rocky shores as it is very effective at colonizing empty space made available by disturbance, has fast growth rates, and inhibits the settlement and growth of other algal species as well as invertebrates (Sousa, 1979). While we did not capture strong differences in *Ulva* sp. among regions in our transect surveys, our experimental plots showed that in the absence of grazer manipulations (i.e., the control plots), *Ulva* sp. was approximately 10 times more abundant in the LS sites compared to the HS sites. Further, previous years show a clear difference in *Ulva* sp. cover between the two regions that was not captured consistently during our transect surveys. Unlike limpets, several species belonging to the genus *Ulva* have demonstrated a wide tolerance for salinities as low as 5 psu (Ichihara et al., 2013) and are therefore widespread in marine and brackish habitats (Rybak, 2018). Our hypothesis that differences in community structure (i.e. the asymmetry in regional abundance of *Ulva* sp.) are indirectly driven by salinity via differences in grazing pressures, not salinity tolerance, was supported by our results. Our lab experiment showed positive net gains in mass and minimal changes to ETR_max_ at a range of salinity levels from 5 to 25 psu, which encompasses most of the salinities experienced at both the HS sites and LS sites during summer. In the HS sites, the absence of limpets in the exclusion experiment allowed *Ulva* sp. to proliferate to abundances that matched that of the low salinity sites. Grazing by gastropods is known to have a direct negative impact on both the abundance and vertical zonation of intertidal foliose algae (Coleman et al., 2006; Hesketh et al., 2021), which is what was documented during our field manipulation. The effect of excluding limpets on algal cover is often strongest in the absence of barnacles, as barnacles increase the habitat structural complexity, impeding the movement of larger grazers (Geller, 1991; Harley, 2006). As our plots were completely cleared at the outset of the experiment, the lack of barnacles may have strengthened the ability of grazers to reduce *Ulva* sp. cover so effectively. Taken together, these results demonstrate that *Ulva* sp. is more common in low salinity regions due to the lack of grazing here, and not via the direct effects of salinity.

In contrast to *Ulva* sp., excluding grazers in the HS sites had the opposite effect on *C. dalli*, whose abundance decreased to match that of the abundance seen in the LS control plots. Limpets can have positive indirect effects on the abundance of *C. dalli* barnacles either by freeing up space for settlement of cyprid larvae that would otherwise be occupied by macroalgae, or by reducing interspecific competition by “bulldozing” the barnacle *B. glandula* (Dayton, 1971; Harley, 2006). Because the presence of grazers did not have a negative effect on *B. glandula* abundance in the control plots in comparison to the exclusion plots, the facilitatory role limpets played in the recruitment of *C. dalli* was likely a result of grazing on algae, and not due to changes in interspecific competition. The former pathway is also supported by experimental limpet exclusions on the rocky shores of Mexico and the Mediterranean, where algal cover increased and the congeneric species *Chthamalus montagui* and *Chthamalus stellatus* subsequently decreased (Arrontes et al., 2004; Benedetti‐Cecchi, 2000; Dungan, 1986). In addition, herbivore presence may also serve as a settlement cue through chemical signals that indicate the presence of suitable habitat for *C. dalli*. Such a mechanism has been shown for *Chthamalus anisopoma*, which has higher settlement in response to the chemical cues of species whose suitable habitat overlaps with its own (Raimondi, 1988).

While our study did not set out to test the mechanisms controlling the abundance and distribution of each species, there are likely other indirect effects of salinity occurring in this system. For example, *M. trossulus* was present at a much higher abundance in the LS sites throughout summer. As *M. trossulus* has a higher salinity tolerance than its main predators, sea stars and dogwhelks (Covernton & Harley, 2020; Held & Harley, 2009; Qiu et al., 2002), its presence in the LS sites and absence in the HS sites is likely due to differences in predation pressure.

Taken together, our results show that indirect effects of abiotic drivers such as hyposalinity can propagate through levels of biological organization, having profound impacts on community structure. The estuarine rocky intertidal system of the Strait of Georgia is driven by osmotic‐related suppression of key grazing species, with cascading effects through trophic interactions that ultimately restructure the entire community. Abiotic conditions that vary in both space and time can lead to dynamic patterns in species distribution and abundance along these spatiotemporal gradients. Climate change is expected to impact salinity regimes in coastal regions both by intensifying the hydrological cycle that impacts precipitation patterns and by shifting the timing and strength of the spring freshet (Held & Soden, 2006). Indeed, both short and long‐term changes to the coastal salinity in the Strait of Georgia have already begun to take place. The 2021 North American heatwave, a climactic event that broke maximum daily temperature records in multiple locations by more than 5°C, resulted in reduced soil water storage capacity, exacerbating autumnal flooding caused by an atmospheric river, and ultimately led to pronounced streamflow and freshwater inputs to coastal systems (White et al., 2023). Additionally, the mean annual salinity on the western boundary of the Strait of Georgia has increased by 3.9 psu since 1935 (Iwabuchi & Gosselin, 2019). While intertidal populations have demonstrated a capacity for local adaptation, the continued expected changes to the global hydrological cycle are likely to alter the structure and composition of coastal communities. Understanding the effects of indirect effects on community dynamics may prove essential to predicting the direction of such change in coastal ecosystems.

## AUTHOR CONTRIBUTIONS

Christopher D. G. Harley initially conceived the idea for the study; Christopher D. G. Harley, Rebecca L. Kordas, and Theraesa Coyle developed the methodology; Theraesa Coyle performed the experiments; Theraesa Coyle and Rebecca L. Kordas conducted the field work; Theraesa Coyle analyzed the *Ulva* lab experiment, Sandra Emry analyzed the field survey data, limpet lab experiments, and the field experiment; Theraesa Coyle wrote the initial version of the manuscript; Sandra Emry wrote the final version of the manuscript. All authors contributed editorial advice.

## CONFLICT OF INTEREST STATEMENT

The authors declare no conflicts of interest.

## Supporting information


Appendix S1.

## Data Availability

Data (Emry et al., 2023) are available in the Borealis repository at https://doi.org/10.5683/SP3/W5JEJM. Code (Emry, 2025) is available in the Open Science Framework (OSF) repository at https://doi.org/10.17605/OSF.IO/XMCNJ.
