# Supplementary material for: Salinity controls rocky intertidal community structure via suppression of herbivory
Source: Ecology. 2025 Dec 8;106(12):e70271. doi: 10.1002/ecy.70271 (PMC12683622; doi:10.1002/ecy.70271)
Supplement: Supplementary file 1 — Appendix S1. [file ECY-106-e70271-s001.pdf]

Appendix S1: Supplementary material

Title: Salinity controls rocky intertidal community structure via suppression of herbivory

Authors: Theraesa Coyle, Sandra Emry, Rebecca L. Kordas, Christopher D.G. Harley

Journal: Ecology

Salinity and tidal emersion tolerance of *Lottia* spp.

Methods

To determine whether the salinity tolerance of limpets is influenced by the periodic emersion from hyposaline conditions experienced during low tides, we conducted a salinity tolerance experiment which incorporated a simulated tidal exposure. Two experiments were performed, one with *Lottia pelta* and the other with *Lottia digitalis*, collected from HS1, Galiano Island, from a salinity of 32 psu. We randomly assigned four limpets to one of twenty-four 1 L Ziploc® containers with mesh walls and two containers to each of twelve 20 L aquaria containing seawater at 30 psu. We randomly assigned four aquaria to each salinity treatment - 30 psu, 20 psu and 10 psu. We then covered each aquaria, provided each with compressed air and placed them inside of a flow through sea water system to maintain a water temperature of 12°C. We lowered salinities by 2.5 psu per day with chilled, dechlorinated freshwater until the desired salinity was reached. To control for water changes, we also performed daily water replacements in treatments that had already reached target salinity, using filtered sea water in place of dechlorinated freshwater.

One randomly selected container within each aquarium was designated as the “intertidal” container, and the other as the “subtidal” container. At 10:00 every morning, we removed the intertidal containers from their aquaria to simulate exposure during low tide. At 18:00 every evening, the containers were placed back inside their aquaria. While out of the water, containers housing the "intertidal" limpets were held at room temperature. Each day, we examined limpets for signs of mortality, including tissue damage, discolouration, and rigidity, and removed dead limpets. The experiment continued for 28 days, and we did not feed limpets during this time.

To test whether emersion during a simulated low tide had an impact on salinity tolerance of either *L. pelta* or *L. digitalis*, we conducted a survival analysis with the *survival* package, version 3.3-1 in R. We modeled the probability of survival with the Kaplan-Meier method, which is a non-parametric method to estimate survival probability from observed survival events.

Tables

Table S1: **Physical characteristics of study sites for intertidal surveys and herbivore manipulation experiment.** Regions include West Vancouver (WV) and the Southern Gulf Islands (SGI). Vertical height refers to height above Canadian chart datum. Slope refers to degrees above the horizontal. Aspect refers the compass direction faced by the substratum.

Site	Region	Latitude	Longitude	Vertical height (m)	Slope (°)	Aspect (°)
LS1 Lions Bay	WV	49.78472	123.4044	3.3	10-22	225-295
LS2 Copper Cove	WV	49.64056	123.4742	3.8	19-37	265-335
LS3 Sharon Cove	WV	49.57306	123.3819	3.3	9-16	95-225
HS1 Hailstorm	SGI	49.65056	123.9697	2.3	10-33	55-75
HS2 Ruckle Park	SGI	49.29250	123.6106	2	4-16	100-135
HS3 Eagle Cove	SGI	49.26750	123.6761	2.4	1-26	165-245

Table S2: Results from a PERMDISP test on species composition data from transect surveys conducted in West Vancouver (LS sites) and the Southern Gulf Islands (HS sites), in the summer of 2011

Df	SS	MS	F	N.Perm	*P*-value
7	0.137	0.020	3.291	999	0.082
184	1.097	0.006			

Table S3: **Contribution of species to the overall Bray-Curtis dissimilarity matrix of invertebrate and algal community composition in low vs. high salinity regions during the summer of 2011, estimated with a similarity of percentages (SIMPER) analysis.** Species which make up 70% of the cumulative contribution are shown, as well as their mean abundance in low and high salinity regions.

Taxon	Avg contribution (%)	Cumulative contribution (%)	Mean abundance low salinity	Mean abundance high salinity
Mytilus trossulus (%)	15.9	19.1	48.7	0.8
Balanus glandula (%)	8.6	29.4	16.1	21.3
Fucus distichus (%)	8.5	39.6	32.7	17.4
Chthamalus dalli (%)	8.1	49.3	0.4	18.8
Petrocelis (%)	4.3	54.5	0.7	2.3
Lottia paradigitalis (no.)	4.1	59.5	2.0	37.6
Barnacle recruits (%)	4.0	64.2	5.5	0.3
Lottia pelta (no.)	3.8	68.8	0.2	0.5

Table S4: **Results from a PERMDISP test on species composition data from grazer manipulation experiments conducted in West Vancouver (LS sites) and the Southern Gulf Islands (HS sites), in the summer of 2011**. Dispersion among the two salinity regions was unequal, as was dispersion among control and exclusion plots.

Df	SS	MS	F	N.Perm	*P*-value
Dispersion by salinity region					
1	0.155	0.155	7.463	999	0.008
82	1.707	0.021			
Dispersion by treatment					
1	0.451	0.451	17.318	999	0.001
82	2.136	0.026			

Table S5: **Contribution of species to the overall Bray-Curtis dissimilarity matrix of invertebrate and algal community composition of a grazer exclusion field experiment in low and high salinity regions**. The specific comparison is shown on the left side of the table, with the species' contribution to average between-group dissimilarity, as well as the mean abundance in each group of the comparison. Mean abundance group a and group b corresponds to the first and second group respectively, reported in the comparison on the left-hand side of the table. Grazer treatments are indicated with a '-' for grazer exclusions, and '+' for grazer controls. Species which make up 70% of the cumulative contribution are shown.

Taxon	Avg contribution (%)	Cumulative contribution (%)	Mean abundance group a	Mean abundance group b
high salinity – vs. high salinity +				
Ulva sp. (%)	18.4	28.9	39.4	5.1
Chthamalus dalli (no.)	15.6	53.4	24.9	153.4
Balanus glandula (no.)	10.8	70.3	1058.4	1138.4
high salinity – vs. high salinity +				
Ulva sp. (%)	18.2	30.6	39.4	57.7
Balanus glandula (no.)	13.8	54.0	1058.4	647.4
Fucus distichus (%)	7.3	66.3	0.0	0.4
Diatoms (%)	6.3	76.9	12.6	0.0
high salinity – vs. low salinity –				
Ulva sp. (%)	17.4	34.0	39.4	49.4
Balanus glandula (no.)	11.7	56.8	1058.4	803.8
Diatoms (%)	6.3	69.1	12.6	0.0
high salinity + vs. low salinity +				
Ulva sp. (%)	24.5	32.5	5.1	57.7
Chthamalus dalli (no.)	15.5	53.2	153.4	38.6
Balanus glandula (no.)	14.3	72.1	1138.4	647.4
high salinity + vs. low salinity -				
Ulva sp. (%)	25.1	36.5	5.1	49.4
Chthamalus dalli (no.)	15.7	59.3	153.4	46.9
Balanus glandula (no.)	11.5	76.1	1138.4	803.8
low salinity + vs. low salinity -				
Ulva sp. (%)	15.8	31.9	57.7	49.4
Balanus glandula (no.)	9.8	51.8	647.4	803.8
Fucus distichus (%)	8.9	69.7	0.4	0.1

Figures:

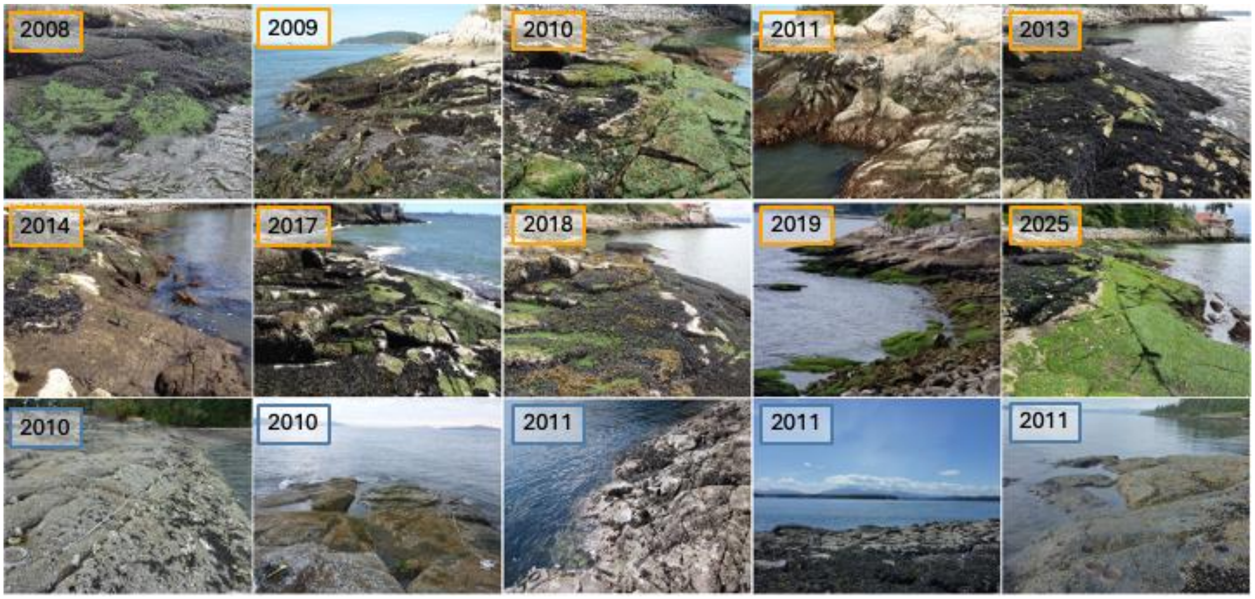

Figure S1: Temporal and spatial variation in *Ulva* sp. coverage. Photos with an orange year indicate this site is in the low salinity region, whereas pictures with a blue year were taken in the high salinity region. Images of low salinity sites taken by Christopher D.G. Harley, images of high salinity sites taken by Theraesa Coyle.

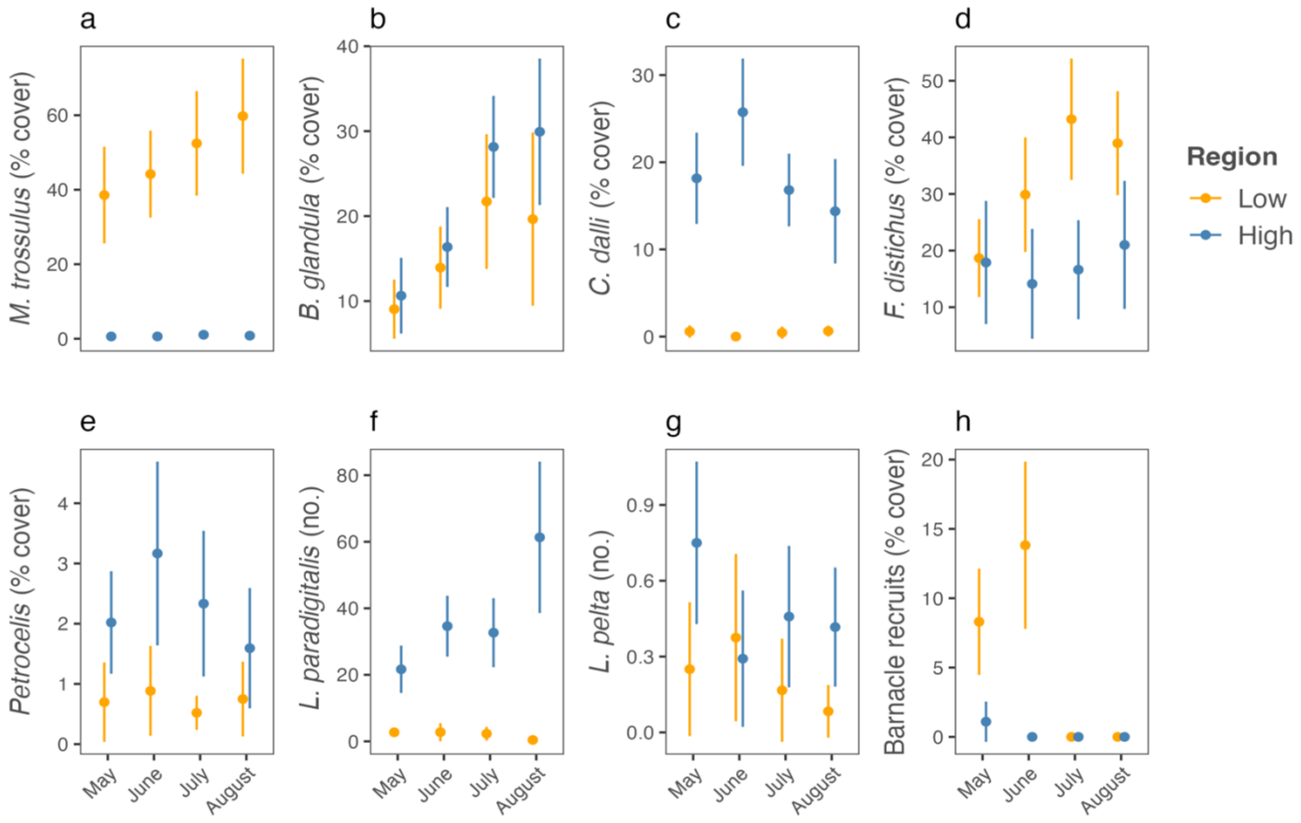

Figure S2: **Mean abundance of species which were key contributors, as identified by a SIMPER analysis, to differences among intertidal communities in a low salinity region, West Vancouver, and a high salinity region, the Southern Gulf Islands during the summer of 2011.** Error bars represent standard error.

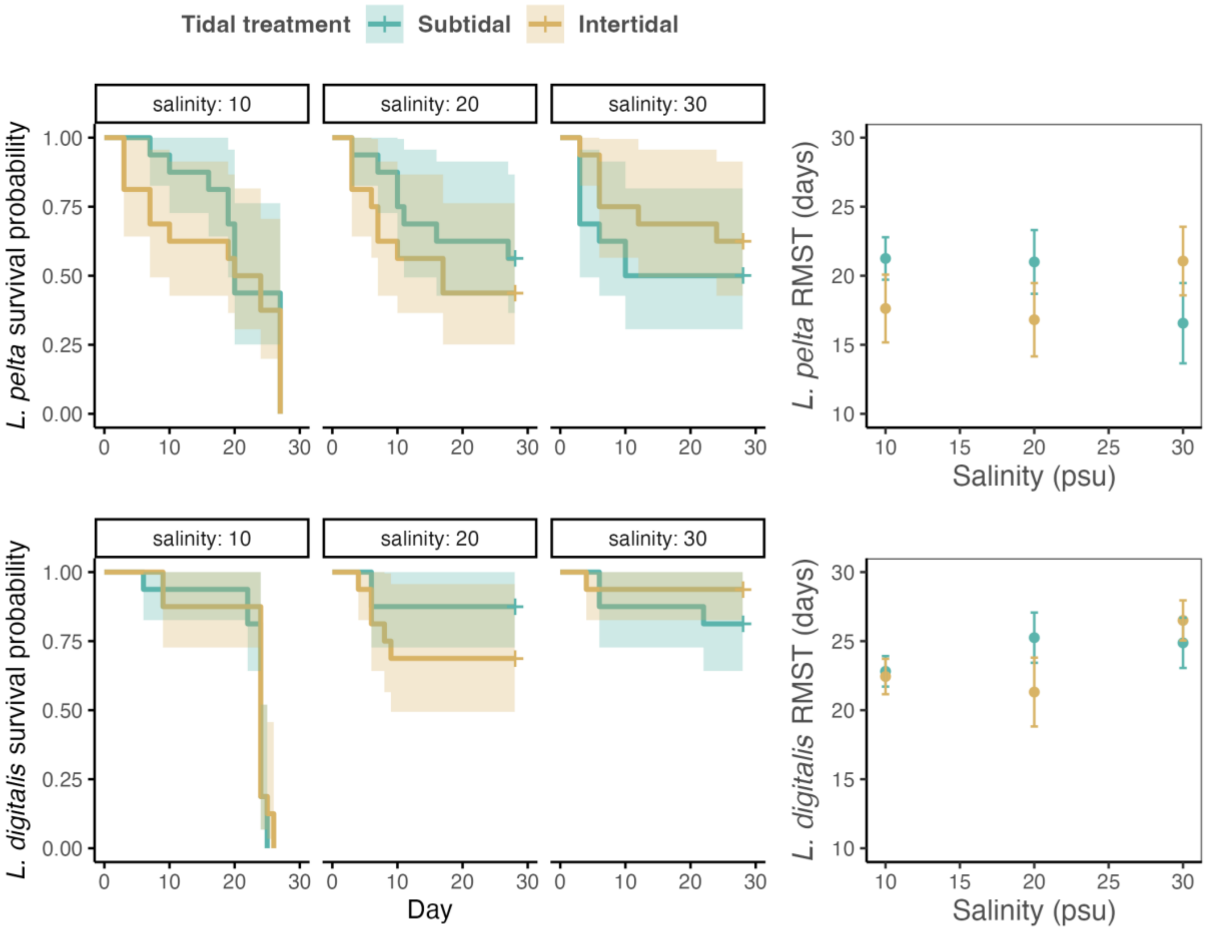

Figure S3: Kaplan-Meier survival curves and restricted mean survival times (RMST) for *Lottia pelta* and *Lottia digitalis*, both collected from HS1 in the Southern Gulf Islands, from a salinity of 32 psu. Limpets were exposed to a salinity of 10, 20 or 30 psu, in either a 'subtidal' treatment (immersed 24 hours/day), or an 'intertidal' treatment (8 hours/day of air exposure). Survival probability is indicated on the y-axis of the Kaplain-Meier survival curves, where 1.0 is equivalent to 100% survival; shaded area indicates 95% confidence intervals. The error bars on the RMST figures indicate standard errors.

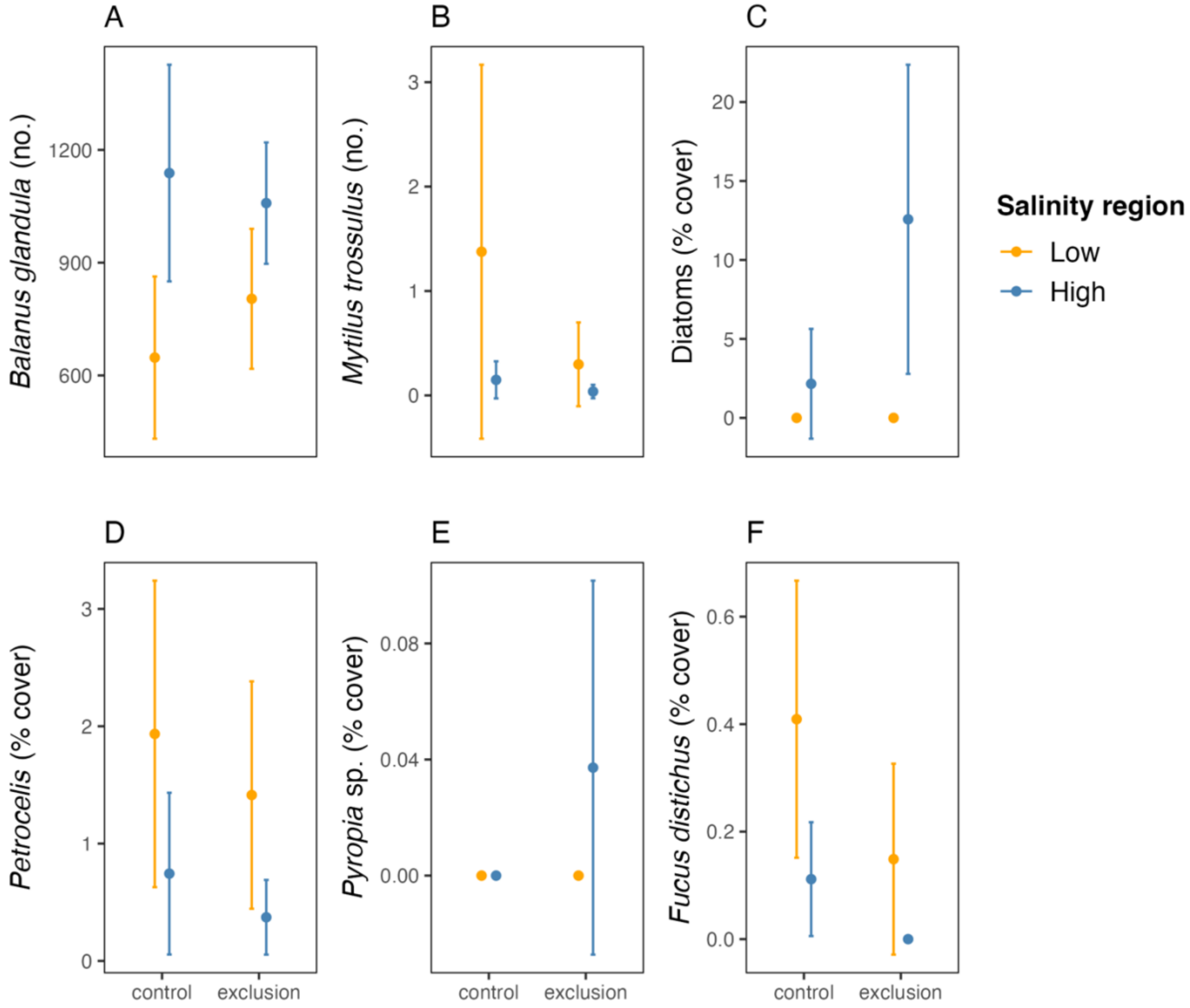

Figure S4: Mean abundance of species which were key contributors, as identified by a SIMPER analysis, to differences among intertidal communities of a grazer exclusion experiment in a low salinity region, West Vancouver, and a high salinity region, the Southern Gulf Islands. Error bars represent standard errors.
